# Supplementary figures and images for: PbTe quantum dots highly packed monolayer fabrication by a spin coating method
Source: PLoS One. 2025 Feb 11;20(2):e0317677. doi: 10.1371/journal.pone.0317677 (PMC11813154; doi:10.1371/journal.pone.0317677)

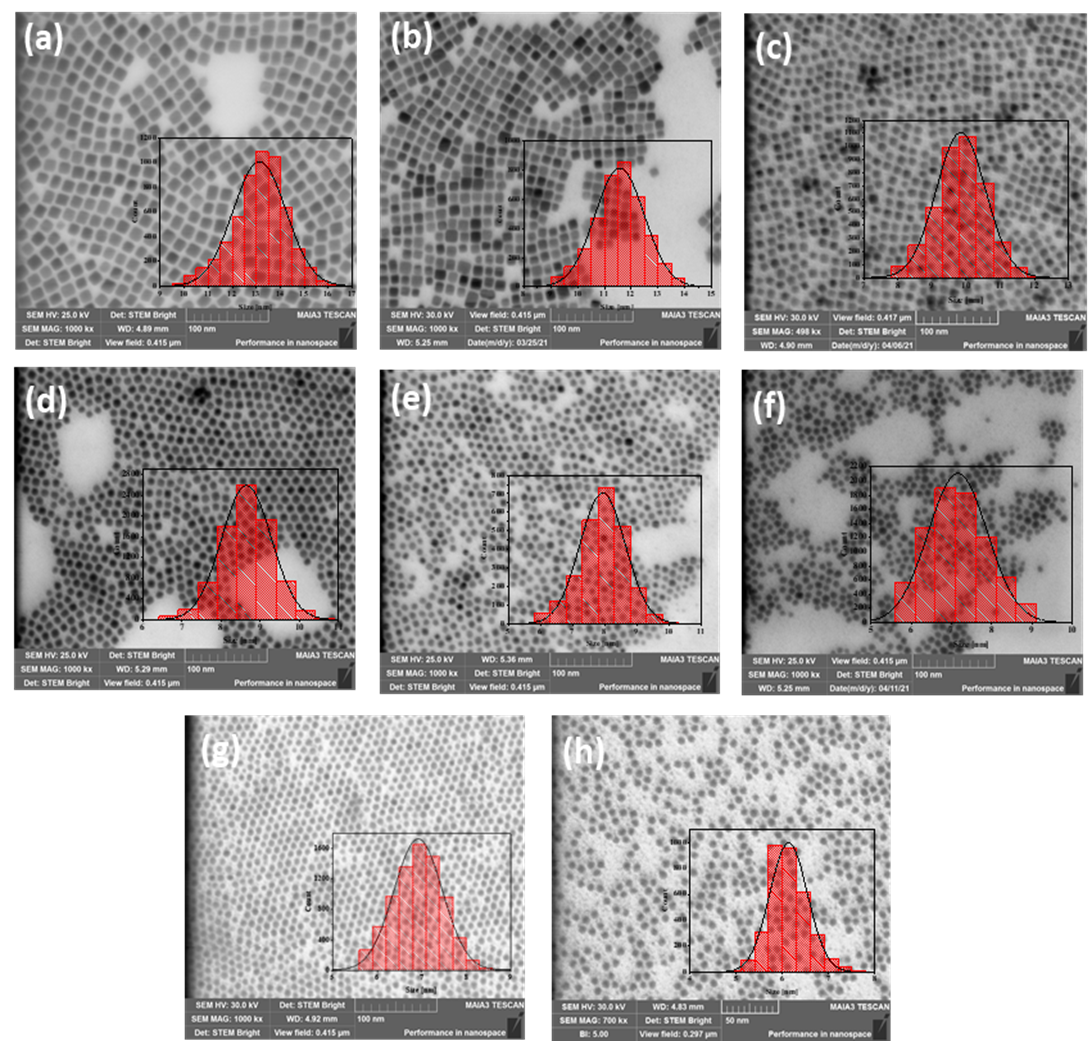

Supplement: S1 Fig — a) 13.2 ± 1.1 nm, b) 11.6 ± 0.9 nm, c) 9.8 ± 0.7nm, d) 8.6 ± 0.6 nm, e) 7.9 ± 0.71 nm f) 7.2 ± 0.7 nm, g) 6.9 ± 0.4 nm, h) 6.1 ± 0.5 nm, used for layer fabrication on aTiO2/ITO glass substrate, by spin coater method. (TIF) [file pone.0317677.s001.tif]

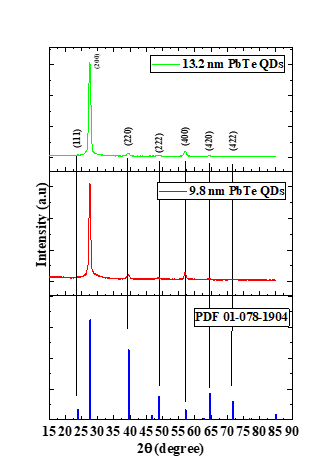

Supplement: S2 Fig — (TIF) [file pone.0317677.s002.tif]

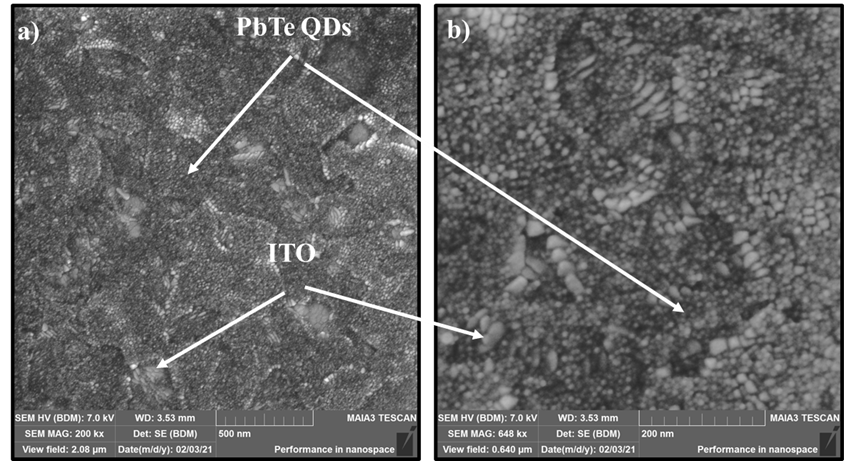

Supplement: S3 Fig — a) low magnification, b) high magnification, prepared by spin coater. Spinning speed: 2000 rpm, spinning time: 30 s, PbTe QDs concentration in hexane–5 mg/ml. (TIF) [file pone.0317677.s003.tif]

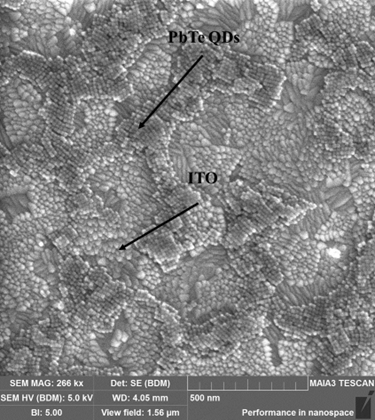

Supplement: S4 Fig — Layer prepared by spin coater with—spinning speed: 2000 rpm, spinning time: 30 s, PbTe concentration in hexane–10 mg/ml. (TIF) [file pone.0317677.s004.tif]

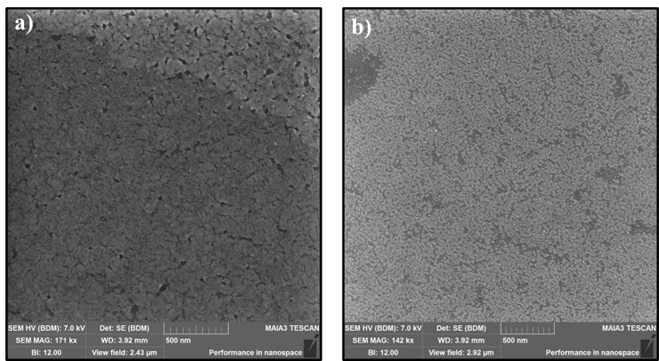

Supplement: S5 Fig — a) 10 mg/ml, and b) 8 mg/ml prepared by spin coater on TiO2/ITO glass substrate. Solvent: hexane, spinning speed: 2000 rpm, spinning time: 30 s. (TIF) [file pone.0317677.s005.tif]

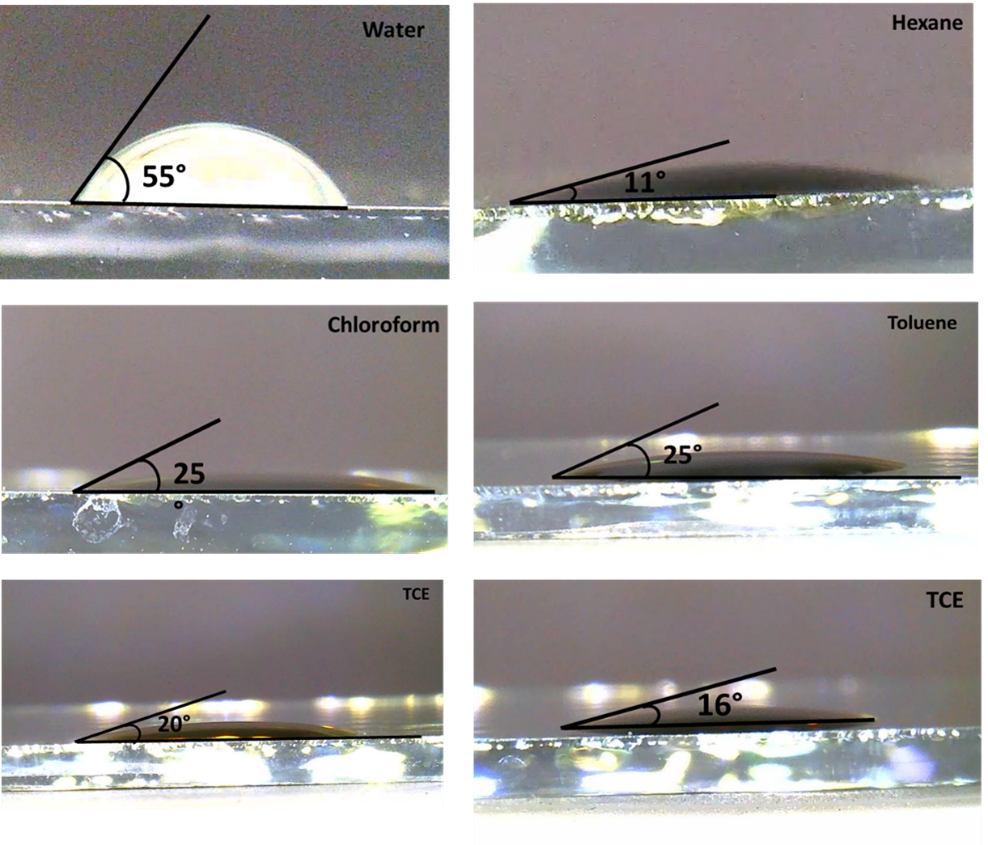

Supplement: S6 Fig — (TIF) [file pone.0317677.s006.tif]

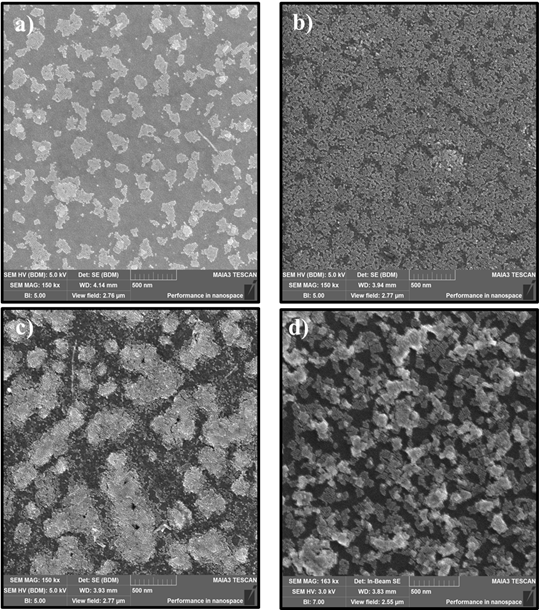

Supplement: S7 Fig — Layers prepared by a spin coater, with a spinning speed of 3000 rpm and spinning time of 30 s, using CHCl3 as a solvent, at different concentrations of PbTe QDs. a) 10 mg/ml, b) 20 mg/ml, c) 25 mg/ml, and d) 30 mg/ml. (TIF) [file pone.0317677.s007.tif]

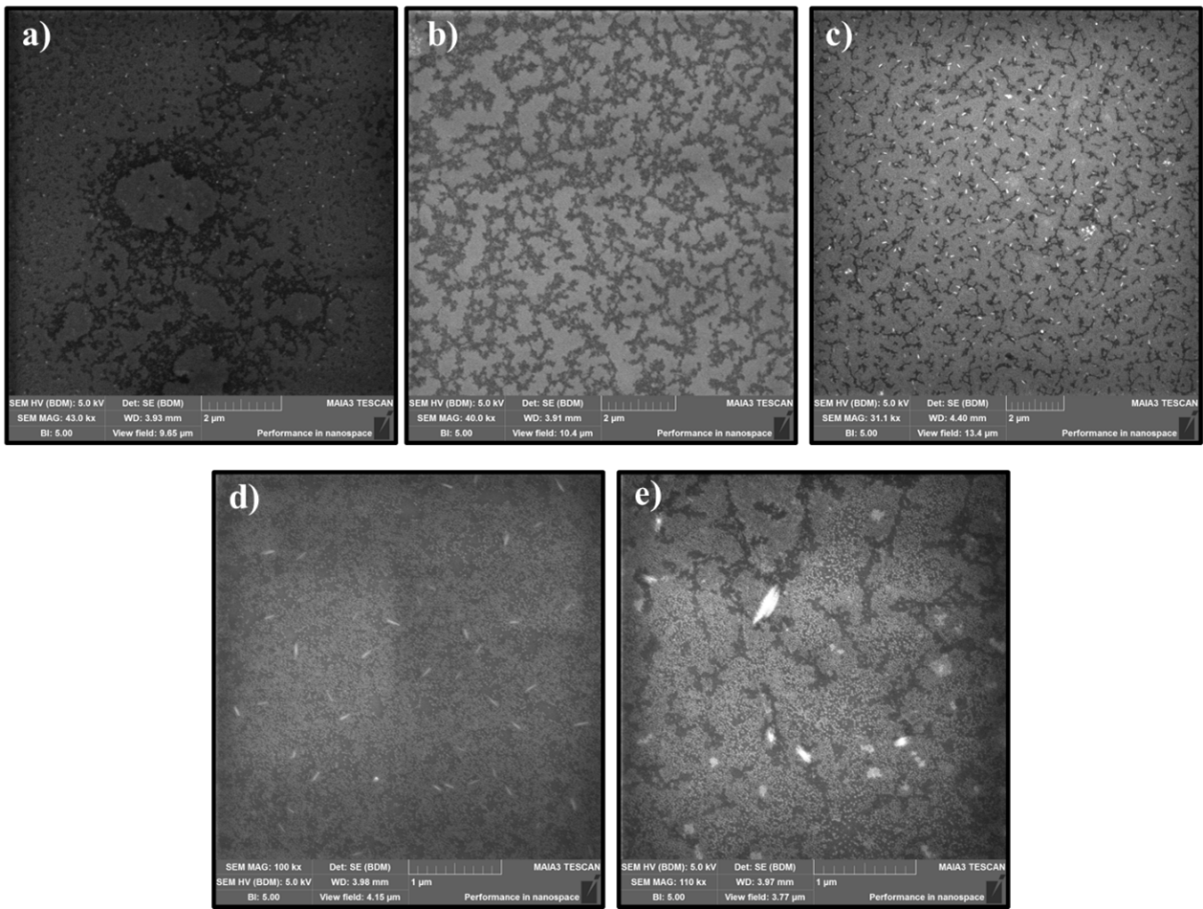

Supplement: S8 Fig — Layers were prepared by a spin coater, spinning time of 30 s, PbTe QDs concentration of 25 mg/ml in CHCl3 with different spinning speeds. a) 1500 rpm, b) 2000 rpm c) 2500 rpm, d) 3000 rpm, and e) 3500 rpm. (TIF) [file pone.0317677.s008.tif]

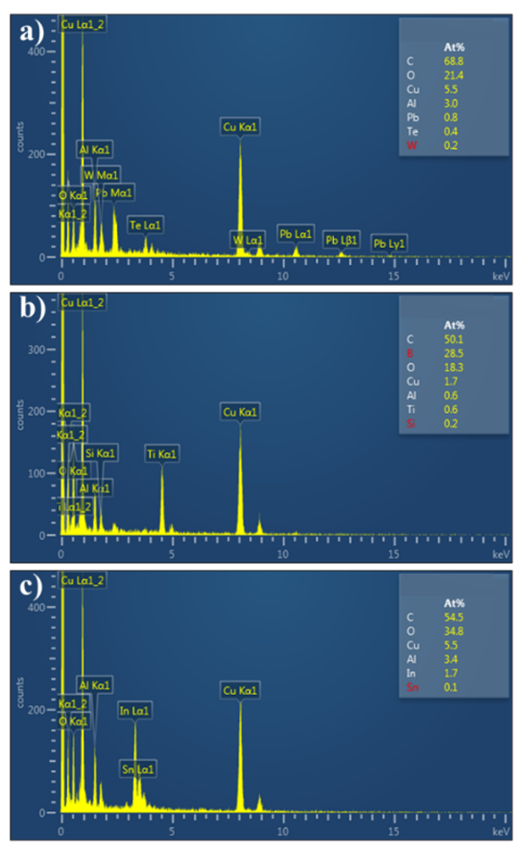

Supplement: S9 Fig — a) PbTe QDs layer, b) TiO2 layer, and c) ITO layer. (TIF) [file pone.0317677.s009.tif]
