# Supplementary material for: PbTe quantum dots highly packed monolayer fabrication by a spin coating method
Source: PLoS One. 2025 Feb 11;20(2):e0317677. doi: 10.1371/journal.pone.0317677 (PMC11813154; doi:10.1371/journal.pone.0317677)
Supplement: S1 Table — (DOCX) [file pone.0317677.s010.docx]

**S1 Table**. **Experimental list/parameters for layers fabrication at various PbTe QDs sizes.**

| **Figure No.** | **PbTe NCs size (nm)** | **Concentration (mg/mL)** | **Solvent** | **Spinning speed (mg/mL)** | **Substrate** |  |
| --- | --- | --- | --- | --- | --- | --- |
|  |  |  |  |  |  |  |
| Figure 2 | 9.8 | 20 | Hexane | 2000 | ITO |  |
| Figure S1 | 6.1 | 5 | Hexane | 2000 | ITO |  |
| Figure S2 | 13.2 | 10 | Hexane | 2000 | ITO |  |
| Figure 3 | 6.1 | 2 | Hexane | 2000 | TiO_2_/ITO |  |
| Figure 4 a | 7.2 | 6 | Hexane | 2000 | TiO_2_/ITO |  |
| Figure 4 b | 8.6 | 9 | Hexane | 2000 | TiO_2_/ITO |  |
| Figure S3 a | 8.6 | 10 | Hexane | 2000 | TiO_2_/ITO |  |
| Figure S3 b | 8.6 | 8 | Hexane | 2000 | TiO_2_/ITO |  |
| Figure 4 c | 9.8 | 12 | Hexane | 2000 | TiO_2_/ITO |  |
| Figure 5 a | 13.2 | 20 | Hexane | 3000 | TiO_2_/ITO |  |
| Figure 5 b | 13.2 | 20 | Toluene | 3000 | TiO_2_/ITO |  |
| Figure 5 c | 13.2 | 20 | Chloroform | 3000 | TiO_2_/ITO |  |
| Figure 5 d | 13.2 | 20 | TCE | 3000 | TiO_2_/ITO |  |
| Figure S4 a | 13.2 | 10 | Chloroform | 3000 | TiO_2_/ITO |  |
| Figure S4 b | 13.2 | 20 | Chloroform | 3000 | TiO_2_/ITO |  |
| Figure S4 c | 13.2 | 25 | Chloroform | 3000 | TiO_2_/ITO |  |
| Figure S4 d | 13.2 | 30 | Chloroform | 3000 | TiO_2_/ITO |  |
| Figure S5 a | 13.2 | 25 | Chloroform | 1500 | TiO_2_/ITO |  |
| Figure S5 b | 13.2 | 25 | Chloroform | 2000 | TiO_2_/ITO |  |
| Figure S5 c | 13.2 | 25 | Chloroform | 2500 | TiO_2_/ITO |  |
| Figure S5 d | 13.2 | 25 | Chloroform | 3000 | TiO_2_/ITO |  |
| Figure S5 e | 13.2 | 25 | Chloroform | 3500 | TiO_2_/ITO |  |
| Figure 6 a | 11.6 | 11 | Hexane | 3000 | TiO_2_/ITO |  |
| Figure 6 b | 11.6 | 11 | Chloroform | 3000 | TiO_2_/ITO |  |
| Figure 6 c | 11.6 | 11 | Toluene | 3000 | TiO_2_/ITO |  |
| Figure 6 d | 11.6 | 11 | Chloroform/Hexane 1:1 | 3000 | TiO_2_/ITO |  |
| Figure 6 e | 11.6 | 11 | Toluene/Hexane 1:1 | 3000 | TiO_2_/ITO |  |
| Figure 7 a | 11.6 | 11 | Toluene/Hexane 1:2 | 1000 | TiO_2_/ITO |  |
| Figure 7 b | 11.6 | 11 | Toluene/Hexane 1:3 | 2000 | TiO_2_/ITO |  |
| Figure 8 a | 6.1@7.9 | 2 | Hexane | 2000 | TiO_2_/ITO |  |
| Figure 8 b | 6.9@6.1 | 6 | Hexane | 2000 | TiO_2_/ITO |  |
| Figure 8 c | 7.9@6.1 | 8 | Hexane | 2000 | TiO_2_/ITO |  |

* [6.1@7.9](mailto:6.1@7.9) meaning- 6.1 QD layer on top of 7.9 QD layer.
