# Supplementary material for: PbTe quantum dots highly packed monolayer fabrication by a spin coating method
Source: PLoS One. 2025 Feb 11;20(2):e0317677. doi: 10.1371/journal.pone.0317677 (PMC11813154; doi:10.1371/journal.pone.0317677)
Supplement: Attachment — Submitted filename: Response to Reviewers.pdf [file pone.0317677.s011.pdf]

Dear Editor and Reviewers,

We greatly appreciate your input and valuable suggestions, which have significantly contributed to improving the quality of the manuscript. We have addressed all your comments and revised the manuscript to align with the journal's style requirements. Additionally, we have provided the relevant raw data to support our findings.

Our point-by-point response is provided below.

Reviewer #1: Ms. Ref. No.: PONE-D-24-40936 PLOS ONE

Comments:
1. In Abstract: The author(s) are advised to avoid vague information and add some key results and numerical findings, mention the type/nature of substrates, processing temperature(s) used for readers. The abstract is detailed but somewhat repetitive, particularly when discussing large-area coating of the underlying substrates, concentrations of coating solutions, etc. Consider condensing the text to avoid redundancy, while still conveying the essential findings.

Answer: Thank you for your feedback. We have revised the abstract to remove redundancy, include key results and numerical findings, and provide details on the substrates, as suggested.

2. Introduction: Please highlights the motivation behind this study, the novelty of your research, and potential contribution to the literature. There are numerous reports in the literature on PbTe QDs.

Answer: Thank you for your comment. We have added the missing information to address the motivation behind this study, the novelty of our research, and its potential contribution to the literature.

(a) The author should justify why they focused on PbTe layer(s) over other important materials, for example, CdTe etc., by highlighting its beneficial optoelectronic features for readers. For example, see Ref1. Ref.1 Optical Materials 155, 115816, 2024. https://doi.org/10.1016/j.optmat.2024.115816

Answer: Thank you for raising this important point. We have added the required information into the introduction section to highlight the beneficial optoelectronic features of PbTe compared to other materials like CdTe. Additionally, we appreciate you providing the interesting reference, which has been helpful in enhancing our discussion.

(b) In lines#42-45, the authors mentioned quantum confinement effect and Bohr excitation radius etc. of materials under study. The authors are advised to elaborate the discussion and provide numerical values of Bohr excitation radius for readers with solid first-hand ref(s).

Answer: Thank you for your valuable suggestion. We have revised the discussion to include the numerical values of the Bohr exciton radius for the materials under study, as requested. Additionally, we have cited recent references to ensure the information is

supported by solid and up-to-date sources. We appreciate your attention to detail, which has helped us improve the clarity and quality of the manuscript.

3. Experimental section:

(a) Give replace the “Instruments” section at the end. (b) Mention clearly how STEM/TEM specimens of QDs were prepared, which solvents were used for QDs dispersion to be drop-casted on Cu-grid. Also mention the grid size/dimensions for readers.

Answer: Thank you for your attention to these details. The manuscript has been revised accordingly.

(c) The authors mentioned that they annealed TiO2 thin films grown on ITO/glass substrates at 550C at heating rate of 5C/min, while the glass softening temperature is well below 550C, how they avoid the microcrack of coating layers at such high temperature?

Answer: Thank you for your question. The glass used in our study is polished soda lime float glass, which has a melting temperature between 1400 to 1600°C. This high melting temperature allows the substrate to withstand annealing at 550°C without risking microcracking in the coating layers. The missing information was added to the manuscript.

4. Results and Discussions.

(a) In lines#185-189, the authors mentioned the different factors that affect the compact and coherent coating of the substrates of interest. For growing nucleation centers, a clean surface of the desired substrates is a very important factor [Ref.2]. The authors did not mention the cleaning or treatment process of the substrate used. Please clarify this information for the readers. Ref.1 MRS Advances 8 (5), 194-200, 2023. https://doi.org/10.1557/s43580-023-00515-3

Answer: Thank you for your comment. The cleaning procedure for the substrates has been clarified and is now detailed in the Experimental section. We appreciate your feedback, which has helped us improve the clarity and completeness of the manuscript.

(b) The scale and numerical values in AFM are not legible. Consider the same comments for all relevant images in the manuscript.

Answer: Thank you for your feedback. The scale and numerical values in the AFM images have been corrected for better legibility, along with all relevant images throughout the manuscript. We appreciate your attention to detail.

(c) In lines#218-226, the authors discovered the substrate surface wetting phenomena by the coating solutions is the crucial factor for coherent and compact layer synthesis. Did the author measure contact angle of the coating solutions/solvents with the substrates used? How author choose suitable solvent(s) for compact layer by spin-coating?

Answer: Thank you for pointing this out. At this stage, we had not yet addressed wetting, and we apologize for the mistaken explanation in the manuscript. We have corrected this section accordingly. Moreover, as you suggested, we conducted contact angle experiments, and the important findings have been added to the manuscript.
Regarding the choice of solvents, we selected them based on two main criteria: their ability to provide good wetting properties on the TiO_2 layer and their effectiveness in dispersing PbTe QDs.

(d) In lines#252-254, the authors wrote, “A tightly packed monolayer of hcp arrays can be detected in this case. The QDs in this layer were highly ordered and monodispersed in their size and shape. However, some amount of surface cracking was identified in the layer.” What did author mean by “hcp arrays”? What was the deposition/processing temperature of thin layer(s)?

Answer: Thank you for your comment. The temperature conditions for the deposition and processing of the thin layers have been clarified in the Experimental section. Additionally, an explanation of "hcp arrays" has been highlighted in the manuscript to provide a clearer understanding for the readers.

(e) Clarify in the Fig. S6. Caption whether images are from S(TEM) images or FE-SEM images. (f) Correct the Fig. 8 caption, Fig.8c.

Answer: Answer: Thank you for your attention to these details. The manuscript has been revised accordingly.

(g) Can author include more magnified area-images of PbTe/TiO2 and TiO2/ITO interfaces with SAED for readers.

Answer: Thank you for your suggestion. We have added a more magnified image of the PbTe/TiO_2/ITO interfaces. EDS experiments for the ITO, TiO_2, and PbTe QD layers are included in the Supporting Information for further clarification. However, we were unable to perform SAED analysis for the cross-sectional images due to the sample's thickness and orientation, which made it challenging to obtain clear diffraction patterns in the cross-sectional view. We appreciate your understanding and hope the additional data provides valuable insights.

(h) In lines#430-433, the authors wrote, “The polarity and hydrophobicity of the surface highly influence the fabricated layer. PbTe QDs are known to be hydrophobic, and for this reason, they create a better layer on hydrophobic TiO2/ITO glass substrate compared to hydrophilic ITO.[56]” The authors are advised to include contact angle of coating solutions/solvents with the substrates of interest to justify this claim.

Answer: Thank you for your valuable suggestion. We understand the importance of including quantitative data to support our claim. We have conducted contact angle measurements for the coating solutions/solvents on the relevant substrates and have added this information to the manuscript to justify the statement. We appreciate your insightful feedback, which has helped us improve the clarity and robustness of our discussion.

5. Conclusion: Please re-write the conclusion part again based on the changes that would be made to the current manuscript and highlight the most important factor(s) that play critical role to attain compact and coherent monolayer of PbTe QDs.

Answer: Thank you for your suggestion. We have re-written the conclusion section to align with the updates made to the manuscript.

Reviewer #2: Manuscript #: PONE-D-24-40936

1. Polish the English language

Answer: Thank you for your feedback. We have made enhanced efforts to polish the English language throughout the manuscript and believe that it is now clear and sufficiently refined to meet your expectations. We sincerely appreciate your input and attention to detail.

2. To improve the manuscript quality, cite for QDs the followings; Quantum Dots: Synthesis, characterization, and optical investigations, IOP Publishing, Bristol, UK. © 2024; Graphene, Nanotubes and Quantum Dots-Based Nanotechnology: Fundamentals and Applications, Elsevier Inc., Netherlands. © 2022; Innovation Discovery 1 (2024) 18-25; Journal of Materials Science: Materials in Electronics 34 (2023) 993-1016; Luminescence 33 (2018) 260-266; Superlattices and Microstructures 88 (2015) 662-667; Canadian Journal of Physics 93 (2015) 1490-1494; Journal of Nanoelectronics and Optoelectronics 10 (2015) 705–710; Materials Science in Semiconductor Processing 39 (2015) 276–282; Solar Energy 115 (2015) 33–39; Renewable Energy 45 (2012) 232-236; Solar Energy 85 (2011) 2283-2287; “Quantum dots-based solar cells for potential application”, in: Mingheng Li (Editor), Energy Systems and Processes: Recent Advances in Design and Control, AIP Publishing, USA. © 2023; “Optical properties of quantum dots”, in: Y. Al-Douri (Editor); Graphene, Nanotubes and Quantum Dots-Based Nanotechnology: Fundamentals and Applications, Elsevier Inc., Netherlands. © 2022

Answer: Thank you for your suggestion. The references you provided were very helpful, and we have cited them to improve the quality of the manuscript. We appreciate your input!

3. Section 2 needs more elaborations

Answer: Thank you for your feedback on Section 2. We weren’t entirely sure which specific details needed correction, but we have added important information that was missing in the previous version of the manuscript. We hope these additions address the points you raised and provide greater clarity.

4. Tables 1-2. Any other data for comparison?

Answer: Thank you for your question. The information presented in Table 1 is directly related to the synthesis of the PbTe QDs. All synthesis parameters remain consistent as outlined in the end of the synthesis section, with only data in the tables differing, and no additional information is needed for this comparison at this stage. Table 2 represents various solvent parameters, and in our opinion, all the necessary information is included. If there is any additional data that you believe could provide further insight into the results, please let us know, and we will be happy to consider it.

5. spherical QDs (6-9 nm) over cubical QDs (10-13 nm). How did you measure?

Answer: Thank you for your question. The QD sizes were measured prior to layer fabrication using HRSEM, as described in the Experimental section. For additional clarity, we have included images and corresponding size distribution histograms in the Supporting Information (Figure S6). We hope this addresses your question.

6. layer with the height of single QD) covering approximately 3 cm^2. How did you measure?

Answer: thank you for your question. You can find an explanation for monolayer formation in our response to question 9. Regarding the coverage of the large area, the ITO substrate used in this study has an area of 3 cm^2. To confirm that a tightly fabricated monolayer was achieved across the entire substrate, we performed area mapping using SEM and AFM analyses. SEM provided high-resolution imaging to verify the uniformity and continuity of the layer, while AFM measurements confirmed the consistency of surface roughness and thickness, validating the monolayer nature of the layer. This combination of techniques ensures a thorough evaluation of the structural and morphological quality across the full 3 cm^2 area. The manuscript has been revised accordingly.

7. Fig. 1. Highlight the thickness

Answer: Thank you for your attention to these details. The manuscript has been revised accordingly.

8. Fig. 2. Highlight the grain size

Answer: Following your suggestion, we have highlighted the grain size in Fig. 2.

9. QDs monolayer. How did you measure?

Answer: The concentration of the QD solution was carefully chosen to ensure monolayer formation. We conducted experiments to identify the optimal concentration, and the results, including SEM images of various experiments with different concentrations, are provided in the manuscript and Supporting Information. These images clearly demonstrate that when the concentration is too low, large holes form in the layer, whereas excessively high concentrations result in multilayers. The differences are distinguishable by the contrast in the SEM images. Once the appropriate concentration was determined, AFM analysis confirmed monolayer formation through the observation of very low surface roughness. Notably, the surface roughness of the TiO_2 substrate was measured to be approximately 1.4 nm, which closely matches the

surface roughness of the QD layer fabricated on top. This further validates the uniformity and monolayer nature of the deposited layer. Additionally, cross-sectional experiments were performed, as shown in Fig. 9, to validate that the layers are monolayers with a height corresponding to a single QD. These combined analyses confirm the successful formation of a QD monolayer. The manuscript has been revised accordingly.

10. XRD is required to provide table and structural properties and crystallite size

Answer: Thank you for your suggestion. We have conducted the XRD experiments and included the structural properties and crystallite size in the Results and Discussion section of the manuscript. We appreciate your feedback in helping us improve the quality of the work.

11. Compare crystallite and grain sizes

Answer: Thank you for your question. We found it important to conduct XRD experiments on two representative morphologies—cubic and spherical—rather than all samples, as the nanocrystal structure was observed to be independent of shape and size. By comparing the crystallite sizes obtained from XRD with the sizes measured using HRSEM, we found that they closely match. This important finding has been added to the manuscript to enhance the discussion and provide clarity. We appreciate your input in guiding us to improve the work.

12. What's about optical studies?

Answer: Thank you for your question regarding optical studies. In this work, our primary focus was on the fabrication of mono- and bilayers under various conditions, with an emphasis on understanding the influence of fabrication parameters on layer quality and morphology. As a result, we did not conduct optical studies of the layers in this study. However, we agree that optical studies could provide valuable insights, particularly for future investigations focusing on the optoelectronic properties of the fabricated layers. We appreciate your suggestion and will consider incorporating such studies in our future research to complement the findings presented here. Thank you for your thoughtful feedback.

13. Pb is a toxic. What's the safety arrangement to do the experiment?

Answer: Thank you for your important question. No special safety arrangements are required for conducting these experiments beyond regular cleaning of the instruments and an annual toxicity test to monitor Pb levels in our lab. Our measurements indicate that the Pb concentrations are very low and remain well within the limits set by international standards for toxicity. We ensure compliance with these guidelines to maintain a safe working environment. Thank you for your consideration.

14. Highlight the advantages of spin coating method

Answer: Thank you for your comment. We have addressed this point in the introduction section, highlighting the advantages of the spin-coating method, including its simplicity, cost-effectiveness, and ability to achieve uniform and high-quality layers

with precise control over thickness. We appreciate your feedback and have worked to ensure this is clearly presented in the manuscript.

15. Section 4 should reflect abstract

Answer: Thank you for your feedback. We have revised Section 4 to ensure it accurately reflects the content of the abstract. The necessary adjustments have been made to improve alignment and clarity. We appreciate your valuable input.

16. 34 of 62 refs is out of date (more than 10 years). Update!

Answer: Thank you for your valuable comment. We have updated the manuscript by removing as many out-of-date references as possible and replacing them with references from the last 10 years to ensure the information is current and relevant. Additionally, we appreciate the materials you provided in your previous comments, which have been instrumental in enhancing the quality of the manuscript. Thank you for your support and constructive feedback.

Thank you for your time and thoughtful consideration.

Dr. Refael Minnes
