# Supplementary material for: PbTe quantum dots highly packed monolayer fabrication by a spin coating method
Source: PLoS One. 2025 Feb 11;20(2):e0317677. doi: 10.1371/journal.pone.0317677 (PMC11813154; doi:10.1371/journal.pone.0317677)
Supplement: Attachment — Submitted filename: Response to Editor 2.pdf [file pone.0317677.s012.pdf]

Dear Editor,

We are pleased to submit the revised version of our manuscript, which has been carefully updated based on your comments and suggestions. We sincerely appreciate the opportunity to improve our work and have made every effort to ensure that the manuscript meets the journal's requirements.
In response to your feedback, we have addressed all comments in detail and made the corrections to the manuscript. To assist in the review process, we have provided a point-by-point response to your comments, outlining the changes made and the rationale behind them. Additionally, we have ensured that the reference list is complete and correct.
We believe that these revisions have enhanced the clarity, coherence, and overall quality of the manuscript. We hope that the updated version meets your expectations and is suitable for publication in PLOS ONE.
Thank you for your time and guidance throughout this process.
Please do not hesitate to contact us if any further modifications or clarifications are required.

Point-by-point response:

1. General Revisions: Please revise the entire manuscript for grammatical accuracy and clarity.
Answer: Thank you for the suggestion. We reviewed the manuscript, identified grammatical mistakes, and corrected them to improve clarity. We appreciate your feedback.
2. Experimental Details: Mention how you obtained square QDs. This information is missing both in the experimental description and in Table 1.
Answer: Thank you for pointing out the missing details regarding the formation of square-shaped quantum dots (QDs). The transition to a cubic shape occurs naturally as the particles grow larger, which is influenced by various parameters, such as the amount of oleic acid (OA) and the growth time. In our experiments, the growth time was kept constant, and the shape variation was achieved by altering the amount of OA. Specifically, higher volumes of OA facilitated the formation of cubic particles due to its role in stabilizing certain crystallographic facets during the growth process.
We have corrected the manuscript accordingly, ensuring that this information is now clearly described in both the experimental section and Table 1. The missing details regarding the cubic QD formation process have been added to provide a more comprehensive understanding of our methodology.

3. Specific Edits: Line 155: Replace the term "purification" with "further cleaning."
Answer: The manuscript has been corrected accordingly.

4. Ligand Exchange: In Section 2.4, specify which ligand was exchanged by PDA. Detail the conditions (e.g., temperature) used during the exchange process. Explain how you verified that the ligand exchange was successful.
Answer: The manuscript has been corrected accordingly, and the missing information has been added.

5. Terminology Consistency: In the abstract, it is stated that round QDs yield better results than cubic ones. However, Section 3 mentions cubic and cuboctahedral QDs. Ensure consistent terminology throughout the manuscript (e.g., rounded/spherical QDs).
Answer: The manuscript has been corrected accordingly.

6. Structural Adjustments: The first paragraph of Section 3 (Results and Discussion) should be merged with the Introduction for better flow and coherence.

Answer: Thank you for your suggestion regarding the structural adjustments. We have carefully reviewed the first paragraph of Section 3 and removed repeated information to improve clarity and flow. Additionally, we have incorporated some of the relevant details into the Introduction to provide better context and coherence. We believe that these changes enhance the overall structure and readability of the manuscript. We hope that the revised version meets your expectations, and we are happy to make further adjustments if necessary.

7. Citations: Review the citations suggested by Reviewer 2. Retain only the strictly necessary ones, as nearly 10% of the citations are to Al-Douri Y.
Answer: Thank you for giving us the opportunity to include only the most relevant references. We appreciate the clarification, as we were initially asked by Reviewer 2 to add around 15 references. While we tried to fulfill this request by including as many references as possible, we also felt it was an inappropriate expectation, as it led to an overrepresentation of certain sources. We have now carefully reviewed the suggested citations and retained only those that support our findings. Thank you for your understanding.

8. Figures and Data Presentation: The images in Figure S1 are unclear. They appear to be in STEM mode within an SEM setup rather than traditional SEM mode. Please clarify and correct this. Correct the sequence of Figures S4 and S5 in the supplementary information. Since layers of 8.6 nm are mentioned earlier in the manuscript, Figure S5 should precede Figure S4. Provide details on how the coverage percentage was measured.
Answer: Thank you for your feedback on the figures and data presentation. We have replaced Figure S1 with a higher-quality version to ensure better clarity and representation.
Regarding the sequence of Figures S4 and S5, we regret to inform you that we cannot change their order, as it reflects the logical progression of our experiments. Initially, we conducted experiments on ITO substrates, as shown in Figure S4, demonstrating the behavior of 13 nm particles. Subsequently, we performed experiments on TiO_2/ITO substrates, as presented in Figure S5, where we illustrate the 8.6 nm QDs layers. Finally, we returned to 13 nm particles, this time preparing the layers on TiO_2/ITO. This sequence accurately represents the chronological and logical flow of our experimental work. Additionally, the missing information regarding the measurement of the coverage percentage has been added to the experimental part of the manuscript for completeness and clarity. We hope these revisions address your concerns and improve the overall presentation of the manuscript.

Best Regards
Dr. Refael Minnes
